# Multiple Functions of D-α-Tocopherol Polyethylene Glycol 1000 Succinate (TPGS) as Curcumin Nanoparticle Stabilizer: In Vivo Kinetic Profile and Anti-Ulcerative Colitis Analysis in Animal Model

**DOI:** 10.3390/pharmaceutics9030024

**Published:** 2017-07-21

**Authors:** Heni Rachmawati, Aditya Trias Pradana, Dewi Safitri, I Ketut Adnyana

**Affiliations:** 1 School of Pharmacy, Bandung Institute of Technology, Ganesha 10, Bandung 40132, Indonesia; dewi.s@fa.itb.ac.id (D.S.); ketut@fa.itb.ac.id (I.K.A.); 2 Research Center for Nanosciences and Nanotechnology, Bandung Institute of Technology, Ganesha 10 Bandung 40132, Indonesia; 3 Department of Pharmacy, University of Surabaya, Kalirungkut Raya, Surabaya 60293, Indonesia; Aditya_trias@staff.ubaya.ac.id

**Keywords:** curcumin, nanoparticle, high pressure homogenization, ulcerative colitis, in vivo kinetic, d-α-tocopherol polyethylene glycol 1000 succinate (TPGS), Biopharmaceutical class system (BCS)

## Abstract

This study was conducted to evaluate the potential benefit of particle reduction down to nanoscale on curcumin, a unique natural active compound facing therapeutic problems due to low solubility and permeability. In addition, the presence of TPGS as a surfactant for multiple functions on curcumin nanoparticle was addressed. Observation was focused on bioavailability enhancement after oral administration and local anti-inflammatory improvement after rectal dosing. Nanonization of curcumin was performed using an up-scalable top down method. Specific animal models were used to study the in vivo kinetic profile and the biological activity of curcumin nanoparticle, compared with curcumin powder. d-α-tocopherol polyethylene glycol 1000 succinate (TPGS)-stabilized curcumin nanoparticle was prepared through homogenization with high pressure of the 1500 bar. An in vivo study was performed after oral administration of the preparations to male healthy Wistar rats, to monitor the plasma kinetic profile of curcumin. The biological activity study was conducted after rectal administration of the preparations in Wistar rats induced by 2,4,6-trinitrobenzene sulfonic acid to develop ulcerative colitis. The curcumin nanoparticle with a size of approximately 200 nm was successfully produced and revealed a better in vivo kinetic profile over the larger size of curcumin mixed with TPGS, with bioavailability (AUC_0-∞_) that was accounted for seven-fold. In addition, the TPGS-stabilized curcumin nanoparticle demonstrated a superior local anti-inflammatory effect in ulcerative colitis, indicated by the shifting of observed parameters close to the healthy status. The tremendously improved anti-inflammatory effect of the TPGS-stabilized curcumin nanoparticle was found with a very low dose. Reducing the particle size of curcumin down to ~200 nm with the presence of TPGS seems to be a promising approach to improving the therapeutic value of curcumin.

## 1. Introduction

Curcumin, a major active constituent found in *Curcuma* sp., is classified in group 4 of BCS (Biopharmaceutical Class System) [1]. This means solubility is a key factor when determining the absorption. In addition, low permeability is a second contribution for its low bioavailability after oral administration. According to Banrida et al., tested curcumin in CaCo2 cells indicated that curcumin is poorly permeable with a Papp (A → B) value of 2.93 ± 0.94 106 cm/s. Papp value in (B → A) study was found out to be 2.55 ± 0.02 106 cm/s, thus ruling out the role of efflux pathways in the low oral bioavailability of curcumin. In addition to poor solubility and permeability, the low bioavailability of curcumin is also worsened by intensive hepatic metabolism to more hydrophilic substances which are inactive [2]. In relation to that study, we also did an in vitro assay of challenging curcumin in liver homogenate and observed rapid degradation after incubation at 37 °C [3]. Curcumin is reported as a potent anti-inflammatory agent [4,5,6,7]; however, its complex problems led to clinical failure when this compound was used. BCS 4 drugs like curcumin are difficult to solve especially in the phase of formulation and delivery. The approach must cover both solubility and absorption enhancements. Incorporation of the permeability enhancer is therefore somehow required.

This report describes two different aims of nanotechnology application on curcumin using TPGS as a stabilizer: to overcome poor oral bioavailability and to evaluate the potential use of curcumin for local anti-inflammation after the rectal route. The latter was performed to study whether the nano-sized curcumin shows preferential accumulation in the inflamed region of the colon and the additional effect of TPGS other than as a particle stabilizer.

The TPGS-stabilized curcumin nanoparticle was developed by top down method using the homogenization technique. An increase in surface area to volume ratio is the mechanism explaining the saturated solubility improvement of the nanoparticle [8].

TPGS, a small molecule, FDA-approved surfactant, was used as a stabilizer based on our previous study that demonstrated good action to prevent particle agglomeration for long term storage in an ambient condition [9]. TPGS has great potential as a drug solubilizer in oral, parenteral, topical, nasal, and rectal/vaginal therapies [10,11]. As reported by many investigators, oral administration of TPGS also functions as a permeation enhancer through the mechanism of P-gp inhibition [8,9,12,13,14]. Furthermore, TPGS is reported to possess antioxidant properties on cellular enzymatic hydrolysis by cytoplasmic esterases that liberate free α-tocopherol, which then localizes in the cell membrane and through free radical quenching protects the membrane from lipid peroxidation and damage [15,16,17]. This is especially interesting in the cases of chronic inflammation of the colon such as ulcerative colitis, considering the pivotal role of oxygen free radicals in the genesis of mucosal damage. Therefore, to demonstrate the beneficial approach on the TPGS-curcumin nanoparticle, we studied the pharmacokinetic profile after oral administration and its effectiveness to treat ulcerative colitis model after the rectal route. The pharmacokinetic study was also performed to confirm our previous study reporting the superior effect of the orally-delivered, TPGS-stabilized curcumin nanoparticle in suppressing carrageenan-induced inflammation in vivo [18]. The rectal route was aimed to observe the local anti-inflammatory effect of the curcumin nanoparticle with the presence of TPGS in the colonic compartment. Eventually, the aim of this bioactivity study on different inflammatory models and the route of administrations of the TPGS-stabilized curcumin nanoparticle is to challenge and convince the potential approach of nanonization. Administration of a far lower dose of the curcumin nanoparticle through the rectal route, reported here, was to obtain the more obvious local effect of curcumin in the inflamed colon. Numerous parameters were evaluated carefully and compared with both TPGS-curcumin suspension and mesalamine as a golden standard for ulceratice colitis treatment.

## 2. Materials and Methods 

### 2.1. Materials

Curcumin from *Curcuma xanthorrhiza* rhizome was purchased from PT Phytochemindo Reksa (Bogor, Indonesia). d-α-tocopherol polyethylene glycol 1000 succinate (TPGS) MW 1500 was purchased from Eastman Chemical Company, South Wales Gwent, UK. Ultra purified water was obtained from a Millipore unit (Millipore GmbH, Darmstadt, Germany). Mesalamine (Salofalk^®^ enemas, Freiburg, Germany), TNBS (Sigma-Aldrich, St. Louis, MO, USA), saline water (Otsuka, Tokyo, Japan), formaline-phosphate buffer 10%, and ethanol pro analysis (JT Baker, Phillipsburg, NJ, USA) were commercially obtained. All other chemicals used in this study were of pharmaceutical grade.

#### 2.1.1. Animal

Pathogen-free male Wi’star rats (6–8 weeks, 150–200 g) (School of Pharmacy, Bandung Institute of Technology, Bandung, Indonesia) were group-housed at the animal house of the School of Pharmacy ITB, with food daily intake of 15 g/rat and allowed unrestricted access to tap water. Rats were allowed to acclimatize for seven days before being used in experiments. Care and experimentation of rats were performed in accordance with institutional guidelines under protocols appropriated by the Institutional Animal Care and Use Committee, Institut Teknologi Bandung, Indonesia, March 2014, Nr. 03/KEPHP-ITB/032014)

### 2.2. Preparation of the TPGS-Stabilized Curcumin Nanoparticle

The TPGS-stabilized curcumin nanoparticle was developed as previously described [9]. Briefly, the curcumin powder (5% *w*/*v*) was suspended in aqueous TPGS solution (1% *w*/*v*) using an Ultra Turrax T25 (Jahnke, Hamburg, Germany). The obtained pre-suspension was subjected to high pressure homogenization (HPH, a Micron Lab 40 APV Deutchland GmbH, Unna, Germany) by applying a pre-treatment of several homogenization cycles at low pressures and subsequently 20 homogenization cycles at 1500 bar.

#### 2.2.1. Particle Size, Particle Size Distribution, and Potential Zeta Measurements

Samples (TPGS-curcumin and the TPGS-stabilized curcumin nanoparticle) were measured by photon correlation spectroscopy (PCS, Delsa™ Nano C Particle Analyzer, Beckman Coulter, Shelburne, VT, USA). Samples were transferred into cuvette and then placed inside the sample holder of the particle size analyser. Once the required intensity was reached, analysis was performed to obtain the mean particle size and PDI of the sample. PDI represents the particle size distribution. The potential zeta of samples was determined in the same way, using the electrophoretic light scattering method (Delsa™ Nano C Zeta Potential Analyzer, Beckman Coulter, Shelburne, VT, USA). PCS measurements were performed at 25 °C, and each sample was analyzed in triplicates.

#### 2.2.2. Scanning Electron Microscopic (SEM) Analysis

The morphology of TPGS-curcumin and the TPGS-stabilized curcumin nanoparticle was observed with SEM. The samples were fixed on a brass stub using double-sided tape and further gold-coated in vacuum by a sputter coater. The analysis was taken at excitation voltage of 10 kV and at 10,000× magnification by using JSM-360LA Scanning Microscope (Jeol, Tokyo, Japan).

### 2.3. In Vivo Kinetic Study of the TPGS-Stabilized Curcumin Nanoparticle after Oral Administration

Male Wistar rats of 200 g weight with an age of 2 months were used in this study. The animals were fasted for 12 h prior to the experiment but given free access for water ad libitum. Animals were divided into two groups (of six rats each), and given TPGS-curcumin suspension or TPGS-stabilized curcumin nanosuspension orally with the same dose of 10 mg/kg BW. Blood sampling of 500 µL was performed through the tail vein at the interval times: 0; 0.25; 0.5; 1; 2; 4; 8; 12, and 24 h after oral administration. The blood samples were placed into heparinized tubes. To obtain plasma, the heparinized blood samples were centrifuged at 12,500 rpm for 5 min. Curcumin in plasma samples were determined by the HPLC method. Prior to HPLC analysis, 200 µL of plasma was added with 80 µL aquabidest, vortexed for 20 s. Ethyl acetate of 480 µL was further added to the plasma-aquabidest mixture, and again vortexed for 30 s. Subsequently, the mixture was centrifuged at 13,000 rpm. The organic phase of 450 µL was taken and vacuum-dried. The residue was re-dissolved in 100 µL of mobile phase, vortexed for 30 s, and was then ready for HPLC analysis. In vivo parameters of curcumin were calculated using computer software Multifit, as previously described, with the 2-compartment model [19].

### 2.4. HPLC System

The Phenomenex^®^ Luna C18 5 µm 100 Å (250 × 4.6 mm) column was used as a static phase. The freshly-prepared mobile phase of phosphate buffer 0.045 M pH 4.5-acetonitrile (45:55) was used with a flow rate of 1 mL/min. The curcumin content was determined using a UV detector at a wavelength of 425 nm. A series concentration of curcumin USP standard was injected to obtain the calibration curve. The curve was found to be linear in the concentration range 0.005–5.00 µg/mL and r = 0.9999. The lower limit of quantification was 0.0025 µg/mL, which could be measured with acceptable accuracy and precision.

Anti-inflammatory Evaluation of the TPGS-stabilized Curcumin Nanoparticle After Rectal Administration.

Ulcerative colitis was used as a model of chronic inflammation to explore the local effect of curcumin in the colonic compartment and the beneficial use of TPGS. To induce colitis, TNBS (2,4,6-trinitrobenzene sulfonic acid) was applied. Twelve hours prior to the induction, animals (5 groups of 6 each) were fasted but given free access for water ad libitum. Then, animals were anesthetized and the TNBS solution in ethanol with a dose of 80 mg/kg body weight was administered via catheter with an outer diameter of 3 mm (Terumo) through the rectum. Subsequently, rats were held by the tail in a vertical position for about 2 min after TNBS administration to avoid TNBS solution leakage. The control group was only given 50% of ethanol using same technique.

To ensure the successful induction, body weight, stool consistency, and the presence of gross blood of feces were observed. The disease activity index (DAI) was calculated by assigning parameters that analog with the clinical manifestation of human IBD (Inflammatory Bowel Disease), as well as those documented by Cooper. Table 1 presents the parameters used for the calculation.

Levels of disease severity were classified as the total of these scores, resulting in total a DAI score ranging from 0 to 12. The scoring ranges are 0 to 2 for normal, 3 to 5 for mild, 6 to 10 for moderate, and 11 to 12 for severe.

Furthermore, daily treatment was given 24 h after induction for 15 days: group a (negative control: healthy, vehicle, water), group b (positive control: TNBS-induced and given only vehicle), group c (TPGS-curcumin suspension 60 mg/kg BW), group d (TPGS-stabilized curcumin nanosuspension 1.8 mg/kg BW), and group e (standard drug-mesalamine 180 mg/kg BW). The dose of TPGS-curcumin and the TPGS-stabilized curcumin nanoparticle was referred to in our previous study [18].

At day 15, all rats were sacrificed, then rapidly dissected, and colons were isolated. The hematocrit of the blood from each rat was measured by using the heparinized-capillary tube. Scoring of the adhesivity of the intestine and macroscopic observation (Table 2) were carried out prior to colon isolation. The isolated colon was gently cleared from feces by multi-step rinsing with cold saline water, then weighed. The colon length was measured and macroscopic damages were observed visually. In addition, the general histological analysis was also performed on the paraffin-embedded colon by a standard eosin-hematoxilyn staining.

### 2.5. Statistical Analysis

Statistical analysis was performed using different ways for pharmacokinetic and activity studies. Pharmacokinetic data were presented as mean ± SD after the two-tailed distribution student *t*-test. Also, the anti-inflammatory effect of the therapies was analysed using one-way ANOVA for parametric data followed by the post-hoc method, and the Mann-Whitney analysis for non-parametric data. *p* value < 0.05 was considered as statistically significant.

## 3. Results

### 3.1. Physical Characteristic of the TPGS-Stabilized Curcumin Nanoparticle

Table 3 presents the physical properties of TPGS-curcumin versus the TPGS-stabilized curcumin nanoparticle.

**Figure 1:**
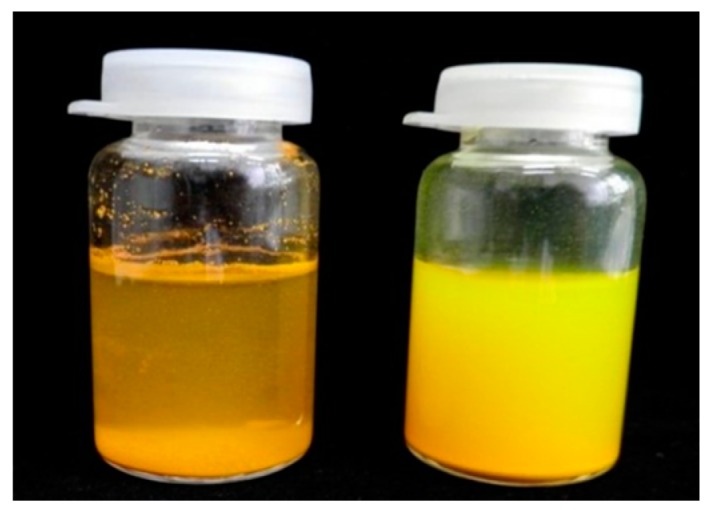
Visual appearance of the suspension of TPGS-curcumin (**left**) and the TPGS-stabilized curcumin nanoparticle (**right**) after 30 min of observation.

The particle size data was confirmed by visual observation of the dispersion characteristic of both TPGS-curcumin and the TPGS-stabilized curcumin nanoparticle (Figure 1). As seen, the fine curcumin nanoparticle showed homogenous dispersion in water as compared to curcumin, which was not completely dispersed and led easily to sedimentation.

**Figure 2:**
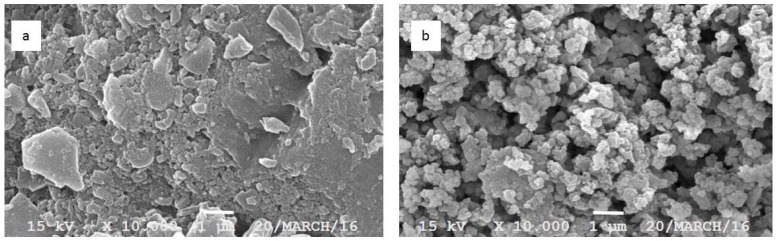
Scanning electron microscopic analysis of TPGS-curcumin (**a**) and the TPGS-stabilized curcumin nanoparticle (**b**). Magnification 10,000×.

The SEM analysis, as depicted in Figure 2, might explain the dispersion characteristic of both forms of curcumin as well as confirming the particle size and polydispersity index, as presented in Table 3. The larger particle size followed by the broad distribution range is suggested as the cause of rapid sedimentation when compared to the fine and more homogenous particle size of the curcumin nanoparticle.

### 3.2. The In Vivo Kinetic Profile of TPGS-Curcumin versus the TPGS-Stabilized Curcumin Nanoparticle

**Figure 3:**
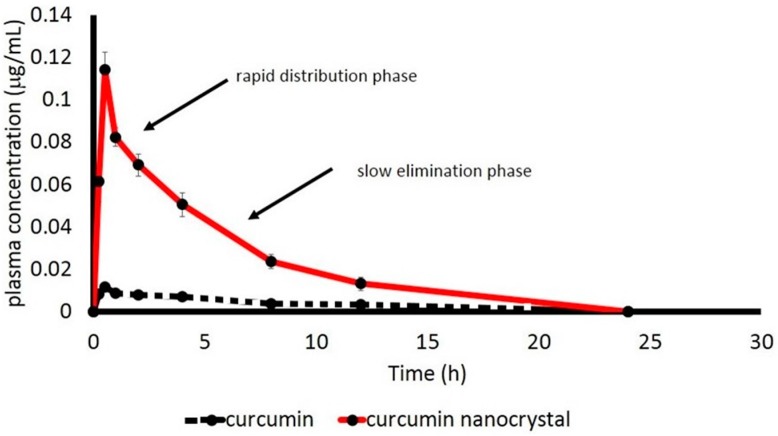
Plasma concentration of curcumin as a function of time after oral administration of TPGS-curcumin suspension and TPGS-stabilized curcumin nanosuspension (10 mg/kg BW) to male healthy Wistar rats (*n* = 6 rats for each group).

The plasma concentration of curcumin after oral administration of both the TPGS-curcumin physical mixture and the TPGS-stabilized curcumin nanoparticle as a function of time is depicted in Figure 3. As clearly seen, curcumin content from the physical mixture is nearly undetected. By contrast, the nano-size of curcumin demonstrated a tremendous increase in bioavailability.

TPGS-stabilized curcumin nanosuspension showed the 2-compartment model, indicated by a rapid distribution phase followed by a slow elimination phase. By computer software Multifit with 2-compartment model analysis, the main kinetic parameters of curcumin are listed in Table 4. The suitability of 2-compartment model fitting was indicated by the value of *R*^2^, which was close to 1.

As described in Table 4, the TPGS-stabilized curcumin nanoparticle demonstrated improved in vivo kinetic parameters. TPGS-curcumin with the larger particle size was absorbed very slowly with a very low amount of nearly undetected in plasma (maximum 0.016 ng/mL after longer *T*_max_ of 1.825 h). Reducing the particle size down to approximately 200 nm followed by the presence of TPGS increased the rate and extent of curcumin absorption as compared to the larger size of curcumin mixed with TPGS. A further consequence is improved bioavailability, which is reflected by an AUC value of approximately seven-fold.

### 3.3. The Anti-Inflammatory Effect of the TPGS-Stabilized Curcumin Nanoparticle after Rectal Administration

The bioactivity study was performed to confirm the potential benefit of the nanonization technique on curcumin, as well as the presence of TPGS. Based on the kinetic data, the dose used for the TPGS-stabilized curcumin nanoparticle was far less than the TPGS-curcumin physical mixture (1.8 mg/kg BW versus 60 mg/kg BW). Curcumin is a well known, potent anti-inflammatory agent [4,5,6,7]; thus, we performed the bioactivity test on the inflammatory model i.e., ulcerative colitis. TNBS-induced colitis is reported by many investigators [22,23,24,25,26], which allows us to study the effect of potential therapy as reported here. To demonstrate the superiority of the TPGS-stabilized curcumin nanoparticle, mesalamine was applied as a standard ulcerative colitis drug. General parameters, as shown in Table 1 and Table 2, which indicated ulcerative colitis development after TNBS induction, were observed. Any reduction on these parameters is suggested as a positive response to the treatment. The data of disease activity index (DAI) is presented in Table 5. As shown, the TPGS-stabilized curcumin nanoparticle demonstrated the fastest recovery (3 days after treatment) as compared to a standard drug mesalamine (4 days). Meanwhile, the TPGS-curcumin physical mixture with a higher dose demonstrated a slow effect. The quantitative anti-inflammatory effect of curcumin, which reduces the disease activity index, is described in Table 5.

**Figure 4:**
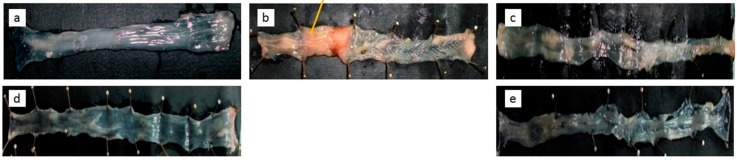
Macroscopic appearance of colon histology: (**a**) negative control: healthy, vehicle, water; (**b**) positive control: TNBS-induced and given only vehicle; (**c**) TPGS-curcumin suspension 60 mg/kg BW; (**d**) TPGS-stabilized curcumin nanosuspension 1.8 mg/kg BW; (**e**) standard drug-mesalamine 180 mg/kg BW. As depicted, chronic inflammation has progressed intensively in the untreated group. This progression has attenuated after treatments, especially using the curcumin nanoparticle. Each group consisted of six rats.

To confirm the disease activity index data visual observation on the colons was performed (Table 6 and Figure 4). As seen in Table 6, the quantitative measurements indicate the anatomic alterations in terms of weight, colon index (the weight ratio of colon to body weight), length, adhesion, lesion, and hematocrit. The deviation to the normal value indicates pathological signs. The normalization of this deviation indicates positive healing. The TNBS-induced animal led to an increase in the value of colon weight, colon index, and lesion number, while reducing colon length, adhesivity, and hematocrit. Treatment with either curcumin or mesalamine shifted those values close to the healthy group, in particular when the animals were treated with a low dose of the TPGS-stabilized curcumin nanoparticle.

The quantitative measurements of the colon are in agreement with the DAI index, which is more reflective of a clinical manifestation.

Similarly, visual observation of the colon (Figure 4) confirmed the successful induction of ulcerative colitis using TNBS, and also successful therapies with curcumin or mesalamine. Again, the nanoform of curcumin demonstrated a better effect, even for a golden standard drug.

**Figure 5:**
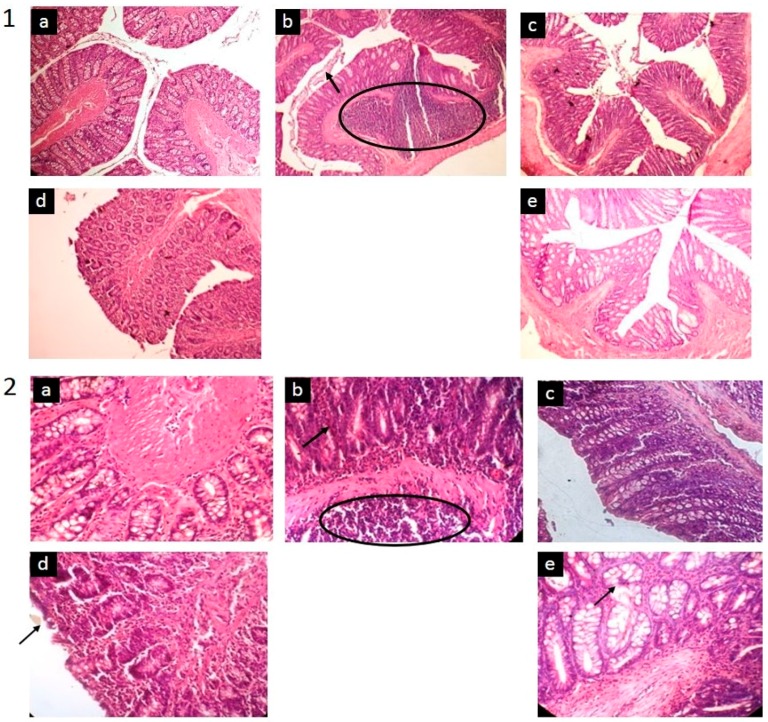
The microscopic appearance of the colon in different groups (*n* = 6 for each): (**a**) (negative control: healthy, vehicle, water); (**b**) (positive control: TNBS-induced and given only vehicle); (**c**) (TPGS-curcumin suspension 60 mg/kg BW); (**d**) (TPGS-stabilized curcumin nanosuspension 1.8 mg/kg BW); (**e**) (standard drug-mesalamine 180 mg/kg BW). As illustrated, there was a massive accumulation of the inflammatory cell in the untreated group (circle) and mucosal disruption in the epithelial tissue (arrow). This sign disappeared after the animals were treated, in particular after they were given the curcumin nanoparticle. Magnification 200× (**1**) and magnification 400× (**2**).

To investigate the colonic lesion that developed after the induction, as well as to confirm the macroscopic data, a standard eosin-hematoxilyn staining was carried out (Figure 5). The clear, normal appearance of the colon mucosa and submucosa on the healthy group was observed. The disease progression shown in group 2 was indicated by the accumulation of infiltrated inflammatory cells into lamina propria.

The normal and healthy colons are characterized by intact and smooth epithelial surfaces, a considerable number of leukocytes, and other cells of the immune system in the lamina propria. In the normal tissue, the surface is lined structurally with simple columnar epithelium that contains several types of cells such as goblet cells, absorptive cells, and enteroendocrine cells.

Administration of TNBS resulted intermediate deterioration in the colonic tissue, indicating the development of an intermediate level of inflammation. This histological analysis confirmed our findings in the DAI (Disease Activity Index). A partial destruction of the mucosa and mononuclear leukocytes infiltrated at high intensity to the sub-mucosal layer of the rat colon was observed in this group. These infiltrations may happen in the other section of the colon and contribute to transmural inflammation and the thickening of colon wall. This evident of intense diffuse inflammatory infiltration was absent after the TNBS-induced groups were treated with TPGS-curcumin, the TPGS-stabilized curcumin nanoparticle, or mesalamine. As clearly shown in Figure 5, the treatment using the TPGS-stabilized curcumin nanoparticle produced a faster recovery reflected by better colonic histology, although a very minor mucosal disruption was still detected. The amelioration of the inflammatory process and the restoration of the epithelial tissue with the regeneration of underlining cells caused by curcumin nanoparticle treatment was better when compared with the higher dose of curcumin (Figure 5.2).

## 4. Discussion

This report describes the development of the TPGS-stabilized curcumin nanoparticle with a detail study to demonstrate the beneficial application of nanotechnology as well as the presence of TPGS to improve the therapeutic value of curcumin, a BCS 4 active compound. Many studies reported wide therapeutic potencies of curcumin in vivo and in clinical phases, as an anti-inflammatory agent [4,5,6,7]. However, clinical failures were faced due to the low bioavailability of curcumin after oral administration. The main factors identified as contributing to this low bioavailability are both poor solubility and permeability. Therefore, any effort improving both parameters is suggested to overcome the clinical use of curcumin. Previously, we reported the successful development of the curcumin nanoparticle stabilized with different agents [12]. Among those five nanoforms, we considered the TPGS-stabilized curcumin nanoparticle for this study to show the multiple benefits of this formulation through in vivo kinetic studies and the improvement in the bioactivity of curcumin. Prior to in vivo studies, the physical parameters (Figure 1 and Figure 2, Table 3) were analyzed to confirm the consistency of the nanoparticle properties. As seen in Figure 2, TPGS prevented particle agglomeration and maintained the particle distribution. The solubility-particle size relationship is well described by the Noyes-Whitney equation [27]. The increased total surface area of the particle due to particle size reduction down to the nano-size is one factor contributing to the improved oral bioavailability of curcumin (Figure 3). The presence of TPGS in the curcumin nanoparticle formulation also played an important role for permeability enhancement, which eventually increased the bioavailability.

d-α-Tocopheryl polyethylene glycol-1000 succinate (TPGS) is a water-soluble source of vitamin E. It is formed by the esterification of polyethylene glycol 1000 with d-α-tocopheryl succinate. Chemically it is a mixture composed principally of the monoesterified polyethylene glycol 1000 (70–87%), the diesterified polyethylene glycol 1000 (<12%), free polyethylene glycol 1000 (<12%), and free tocopherol (<1.5%) [28].

TPGS or vitamin E-conjugated PEG 1000 has received much attention as a permeability enhancer for several poor bioavailability active compounds, clinically demonstrating that TPGS can enhance the absorption of the highly lipophilic drug cyclosporine [29]. The mechanism explaining the permeability enhancement is reported through P-glycoprotein (P-gp) inhibition. TPGS is a more effective P-gp inhibitor than many related excipients with surfactant properties [13]. Vitamin E-containing polyethylene glycol 1000 exhibits greater P-gp inhibition than analogs containing polyethylene glycol 2000 to 6000 [14]. Moreover, several reports describe the beneficial use of TPGS as an excellent antioxidant due to the d-α tocopherol component which is released enzymatically in the cellular membrane. The released d-α tocopherol subsequently protects membrane lipids from peroxidation by scavenging not only chain-carrying peroxyl radicals but also singlet oxygen and the superoxide anion radicals inducing the mucosal damage. This is, in turn, contributing in the regression of inflamed tissues [15,16,17].

Previously, we reported the superior anti-inflammatory effect of the TPGS-stabilized curcumin nanoparticle in an animal induced with carrageenan [18]. We found better a anti-inflammatory effect after oral administration of a low dose of TPGS-stabilized curcumin nanosuspension (60 times lower than the dose of TPGS-curcumin suspension). The pharmacokinetic data we describe in the present study clearly explains this phenomenon.

Thus, the multiple functions of TPGS in the curcumin nanoparticle obviously improved not only the dissolution and absorption of curcumin but also its anti-inflammatory effect. The latter is confirmed when the TPGS-stabilized curcumin nanoparticle was given through the rectal route for local anti-inflammation in TNBS-induced ulcerative colitis. TNBS-colitis in rats was originally reported as a model to induce long-lasting inflammation and ulceration of the rat colon, and the reproducibility was highlighted [23]. This model is characterized by oxidative stress and mucosal infiltration by polymorphonuclear cells, as also seen in this study.

Chronic inflammation is associated with the alteration of cell-signaling pathways, which result in increased levels of inflammatory markers, lipid peroxides, and free radicals, causing cell damage and eventually leading to the clinical symptoms of disease. Histological analysis, as depicted in Figure 5.2, obviously reveals that TNBS yields submucosal infiltration of leukocytes, which have been suggested as being responsible markedly for the tissue damage. Leukocytes are contributors of inflammatory mediators and a major source of ROS in inflamed colon mucosa, so that the infiltration of these cells into mucosa causes significant tissue damage and dysfunction of the colon mucosa [30]. The visual manifestation of this microscopic data was observed in our study, including the DAI (Table 5) and clinical symptoms (Table 6, Figure 4). As demonstrated, treatment of TNBS-induced colitis with either the TPGS-curcumin physical mixture, the TPGS-stabilized curcumin nanoparticle, or mesalamine repaired the colon damages significantly. The presence of TPGS is suggested as also being involved synergistically with curcumin to restore the inflamed colon. However, the disease animal was obviously healed when given the TPGS-stabilized curcumin nanoparticle, although the dose was very low. So, the superior local anti-inflammatory effect of the TPGS-stabilized curcumin nanoparticle seems to be a combination effect of TPGS and nanonization. As described by Beloqui et al. [31], the nano-sized drug represents a promising approach for colon targeting, mostly due to preferential accumulation in the inflamed regions of the colon. The increased mucus production, mucosal surface alterations, crypt distortions, and ulcers lead to a higher accumulation of the nanoparticle in the inflamed colonic region than in healthy tissues.

To our knowledge, this is the first report discussing ability of the TPGS-curcumin nanoparticle to improve the in vivo kinetic profile of curcumin, as well as the local therapeutic benefit to the chronic inflammatory model. The dual approaches of using TPGS and the reduction of the curcumin particle to nanoscale are suggested as a promising form for curcumin to treat chronic diseases associated with tissue damage.

## 5. Conclusions

Vitamin E TPGS exhibits great potential as an excipient due to its multiple functions in drug delivery. The curcumin nanoparticle, stabilized with vitamin E TPGS, preserved the curcumin particle distribution in the nano-range, which is in turn improving oral bioavailability accounted for seven-fold as compared to the larger size of curcumin. Through both mechanisms of particle size reduction and enhanced permeability, the rate and the extent of absorption of the curcumin nanoparticle were significantly increased, as indicated by the better in vivo kinetic parameters such as *T*_max_, *C*_max_, and AUC_0-∞_. In addition, rectal administration of the TPGS-stabilized curcumin nanoparticle with the lower dose also demonstrated a better local effect to suppress TNBS-induced ulcerative colitis in the animal model. Preferential accumulation of the nano-size of curcumin in the inflamed colon and the co-presence of TPGS with the excellent antioxidant are suggested as important points for this superior effect. Thus, the TPGS-stabilized curcumin nanoparticle exhibits combination effects and is a promising form to improve the therapeutic value of curcumin.

## Figures and Tables

**Table 1:** Standard Observation Parameter during Evaluation of Ulcerative Colitis [20].

Parameter	Score	Observation
Stool concistency	0	Normal
	2	Loose stool
	4	Watery stool
Bleeding on feces	0	None
	2	Slight bleeding
	4	Gross bleeding
Weight loss	0	No weight loss
	1	1–5% weight loss
	2	5–10% weight loss
	3	10–15% weight loss
	4	>15% weight loss

**Table 2:** A Standard Macroscopic Parameter of Ulcerative Colitis [21].

Parameter	Score	Disease Status
Adhesivity	0	None
	1	Minor (easily separated)
	2	Major
Colon weight	-	Linear with the disease stauts
Colon length	-	Linear with the disease status
Number of lesion	-	Linear with the disease status

**Table 3:** Physical characteristic of TPGS-curcumin versus the TPGS-stabilized curcumin nanoparticle.

	Size (µm)	Polydirsity Index	Potential Zeta in Water (mV)
TPGS-curcumin	>7	>1	−1.57 ± 0.011
TPGS-stabilized curcumin nanoparticle	0.21 ± 0.008	0.44 ± 0.020	−12.8 ± 1.052

Measurement was done in triplicates.

**Table 4:** The kinetic parameters of TPGS-curcumin suspension versus TPGS-stabilized curcumin nanosuspension after oral administration of 10 mg/kg BW in male healthy Wistar rats.

Sample	T1/2-1(h)	T1/2-2(h)	AUC (µg·h/mL)	*T*_max_ (h)	*C*_max_ (mg/L)	*R*^2^
TPGS-Curcumin suspension	0.019 ± 0.003	1.359 ± 0.201	0.084 ± 0.05	1.825 ± 0.052	0.016 ± 0.006	0.927
TPGS-stabilized curcumin nanosuspension	0.142 ± 0.041	2.9623 ± 0.013	0.562 ± 0.112	0.718 ± 0.210	0.090 ± 0.002	0.993

*n* = 6 rats for each group.

**Table 5:** The disease activity index (DAI) of the animals after being treated with TPGS-curcumin, the TPGS-stabilized curcumin nanoparticle, and mesalamine for 15 days.

Day	Group				
	a	b	c	d	e
1	0.50 ± 0.55	4.17 ± 1.72	3.67 ± 2.58	3.67 ± 1.86	5.33 ± 2.16
2	0.33 ± 0.82	4.33 ± 2.73	3.83 ± 2.99	3.50 ± 2.74	6.00 ± 2.10
3	0	4.17 ± 2.23	3.50 ± 2.81	1.33 ± 1.97	4.50 ± 0.84
4	0	4.50 ± 2.95	2.17 ± 2.14	1.33 ± 1.97	1.67 ± 0.82
5	0	5.00 ± 3.22	2.67 ± 1.75	0.83 ± 1.60	2.17 ± 1.83
6	0	5.00 ± 2.97	2.33 ± 2.88	0.50 ± 0.84	2.67 ± 1.21
7	0	5.00 ± 2.19	3.17 ± 3.54	1.83 ± 1.94	1.50 ± 1.52
8	0	5.00 ± 2.00	2.00 ± 2.61	1.17 ± 2.04	1.50 ± 1.22
9	0	4.83 ± 2.04	1.8 ± 1.83	0.17 ± 0.41	1.50 ± 1.76
10	0	4.67 ± 2.07	1.17 ± 1.33	0.67 ± 1.63	1.00 ± 1.10
11	0	3.00 ± 1.10	0.50 ± 0.84	0.17 ± 0.41	0.67 ± 1.03
12	0	3.17 ± 1.33	0.50 ± 0.84	0	0.83 ± 1.60
13	0	2.50 ± 1.22	0.33 ± 0.52	0.17 ± 0.41	0.17 ± 0.41
14	0	2.33 ± 0.82	0.33 ± 0.82	0	0.83 ± 1.60
15	0	2.33 ± 0.82	0.67 ± 1.21	0	1.00 ± 1.67

Group a (negative control: healthy, vehicle, water); group b (positive control: TNBS-induced and given only vehicle); group c (TPGS-curcumin suspension 60 mg/kg BW); group d (TPGS-stabilized curcumin nanosuspension 1.8 mg/kg BW); group e (standard drug-mesalamine 180 mg/kg BW). Each group consisted of six rats.

**Table 6:** Macroscopic Observation of the Colon, Adhesion Scoring, and Hematocrit Measurement of Treated Groups.

Group (*n* = 6)	Colon Weight (g)	Colon Index (%)	Colon Length (cm)	Lesion Number	Adhesivity	Hematocrit
a	1.33 ± 0.30 *	0.67 ± 0.13 *	13.42 ± 1.32 *	1.33 ± 2.16 *	2.33 ± 1.97	0.39 ± 0.02 *
b	1.90 ± 0.08	0.91 ± 0.11	8.58 ± 1.49	20.33 ± 2.73	1.83 ± 0.41	0.27 ± 0.06
c	1.61 ± 0.12 *	0.77 ± 0.03 *	11.07 ± 1.99 *	14.17 ± 1.72 *	0.33 ± 0.52	0.38 ± 0.03 *
d	1.36 ± 0.26 *	0.69 ± 0.13 *	11.47 ± 1.12 *	12.17 ± 2.48 *	0.33 ± 0.52	0.43 ± 0.03 *
e	1.42 ± 0.06 *	0.69 ± 0.11 *	11.08 ± 0.47 *	15.16 ± 3.43 *	1.40 ± 0.55	0.37 ± 0.05 *

Group a (negative control: healthy, vehicle, water); group b (positive control: TNBS-induced and given only vehicle); group c (TPGS-curcumin suspension 60 mg/kg BW); group d (TPGS-stabilized curcumin nanosuspension 1.8 mg/kg BW); group e (standard drug-mesalamine 180 mg/kg BW). * significantly different as compared to untreated group (b) (*p* < 0.05).
